# 经支气管超声引导针吸活检在肺癌诊断及分期中的初步应用

**DOI:** 10.3779/j.issn.1009-3419.2010.05.11

**Published:** 2010-05-20

**Authors:** 鸿 胡, 海泉 陈, 贤 周, 波 平, 丽青 冯, 建华 周, 晓阳 罗, 富 杨, 挺 叶, 磊 沈

**Affiliations:** 1 200032 上海，复旦大学上海医学院附属肿瘤医院胸外科 Department of Thoracic Surgery, Cancer Hospital, Shanghai Medical College, Fudan University, Shanghai 200032, China; 2 200032 上海，复旦大学上海医学院附属肿瘤医院病理科 Department of Oncology, Cancer Hospital, Shanghai Medical College, Fudan University, Shanghai 200032, China

**Keywords:** 经气管镜超声引导针吸活检术, 肺肿瘤, 诊断, 分期, Endobronchial ultrasound-guided transbronchial needle aspiration, Lung neoplasms, Diagnosis, Staging

## Abstract

**背景与目的:**

经支气管超声引导针吸活检（endobronchial ultrasound-guided transbronchial needle aspiration, EBUS-TBNA）是近年来新的纵隔淋巴结定性诊断方法。本研究旨在探讨其应用于肺癌诊断及分期中的价值。

**方法:**

2009年4月1日-2010年2月8日，75例本院胸部增强CT提示肺内占位伴多发纵隔淋巴结肿大患者接受EBUS-TBNA检查。以最终病理诊断为金标准，检验EBUS-TBNA诊断肺癌纵隔淋巴结转移的准确性、敏感性、特异性、阳性及阴性预测值，并判断其用于非小细胞肺癌N分期的准确率。

**结果:**

75例患者共计穿刺177组病灶区域，平均穿刺2.4组/例。75例患者，组织病理标本送检率为49.33%。以177组穿刺区域计算，组织病理标本送检率为28.81%。75例患者EBUS-TBNA诊断准确率为98.66%，敏感性为98.43%，特异性为100.00%，阳性预测值为100.00%，阴性预测值为91.67%。以177组穿刺区域计算EBUS-TBNA诊断准确率为96.05%，敏感性为95.10%，特异性为100.00%，阳性预测值为100.00%，阴性预测值为82.93%。上述指标除敏感性（*P*=0.435）外，均高于CT检查（*P* < 0.05）。73例可行N分期患者中，19例（26.03%）患者EBUS-TBNA检查后出现分期改变。

**结论:**

EBUS-TBNA准确率较高，创伤小，是用于肺癌诊断及分期的较好方法。

## Abstract (en)

**Background and objective:**

It has been proven that endobronchial ultrasound-guided transbronchial needle aspiration (EBUS-TBNA) was a new minimally invasive method in the diagnosis for mediastinal lymph nodes. The aim of this study is to evaluate the diagnostic and staging yield of EBUS-TBNA for lung cancer.

**Methods:**

Seventy-five patients with tumors and enlarged mediastinal lymph nodes found by CT underwent the diagnosis by endobronchial ultrasound-guided transbronchial needle aspiration (EBUS-TBNA) from April 1, 2009 to Febuary 8, 2010. The sensitivity, specificity, positive predictive value, negative predictive value and diagnostic accuracy of EBUS-TBNA were evaluated.

**Results:**

Seventy-five patients with 177 lymph node groups (2.4 groups in average) were studied. Histopathological samples were found in 49.33% patients and in 28.81% groups. Overall sensitivity, specificity, positive predictive value, negative predictive value and diagnostic accuracy of EBUS-TBNA for diagnostic were 98.43%, 100.00%, 100.00%, 91.67% and 98.66%, respectively, in per patient analysis and were 95.10%, 100%, 100.00%, 82.93%, and 96.05%, respectively, in per group analysis, higher than CT examination (*P* < 0.05) expect for sensitivity (*P*=0.435). Staging changed in 19 (26.03%) patients after EBUS-TBNA.

**Conclusion:**

EBUS-TBNA proved to be a safe procedure with a high yield for the diagnosis of lung cancer.

肺癌是威胁人类健康的主要恶性疾病之一，其发病率及死亡率在我国不断上升^[1, 2]^。准确的诊断及治疗前分期是制定综合治疗方案及推测预后的关键。近年来经支气管超声引导针吸活检（endobronchial ultrasoundguided transbronchial needle aspiration, EBUS-TBNA），以其操作简单、微创、涉及纵隔淋巴结区域广、可重复强的优势，在肺癌分期中逐渐得到广泛应用，已经在一定程度上有取代纵隔镜检查这一传统“金标准”分期方法的趋势^[3, 4]^。本院曾经报道一组20例患者EBUS-TBNA检查结果，纵隔淋巴结定性诊断总体准确率为90.00%（其中上皮性癌诊断准确率为100.00%），敏感性为84.62%，特异性为100.00%，阳性预测值为100.00%，阴性预测值为77.78%^[5]^。本文拟对EBUS-TBNA应用于肺癌的诊断及分期应用做进一步探讨，以期对临床有所帮助。

## 1 材料与方法

### 1.1 患者资料

2009年4月1日-2010年2月8日，117例患者于本院接受EBUS-TBNA检查。其中75例本院胸部增强CT提示肺内占位伴多发纵隔淋巴结肿大，初诊肺癌不能排除[男性57例，女性18例，中位年龄59岁（24岁-84岁）]。上述患者均无重要脏器功能障碍等手术禁忌症，术前气管镜检查气管内均未见病变，均无术前病理诊断。

75例患者中，可疑原发病灶位于左肺30例，右肺43例，另有1例患者为双肺弥漫病灶，1例为纵隔型肺癌。实际接受EBUS-TBNA进行分期患者共计73例，其中临床诊断N1为10例，N2为40例，N3为23例。

上述75例患者中，临床诊断Ⅳ期16例，包括脑转移4例，以MRI为确诊依据。其余包括多发骨转移8例，脾脏转移1例，双肺内转移3例，均以CT为确诊依据。其中1例曾行脑内病灶放疗及全身化疗。

### 1.2 检查设备及穿刺方法

检查使用超声内窥镜型号BFUC206F-OL8，超声图像处理设备型号EU-C2000，穿刺针为型号NA-201SX-4022。上述产品均为Olympus公司产品。

患者平卧位，静脉麻醉，经口插入气管镜，采用水囊法^[5]^穿刺。

### 1.3 病理检查

穿刺标本常规送细胞学病理检查，部分送组织病理学检查。

送检组织采用常规HE染色方法进行检测，如EBUSTBNA细胞病理学取得恶性证据，则以该结果为确诊依据；如EBUS-TBNA细胞病理学结果未见恶性证据，则同期给予纵隔镜检查，并以纵隔镜检查结果为最终确诊依据。

### 1.4 观察指标

临床诊断淋巴结转移依照胸部增强CT，如纵隔淋巴结呈类圆形，且直径≥10 mm记为转移（即阳性），反之为阴性。

EBUS-TBNA穿刺标本中，如病理证实取得病灶内组织为穿刺成功，以最终确诊结果为金标准。恶性诊断记为阳性结果，良性诊断记为阴性结果。与金标准对比，检验EBUS-TBNA诊断肺癌纵隔淋巴结转移的准确性、敏感性、特异性、阳性及阴性预测值。并判断其用于非小细胞肺癌N分期的准确率。

### 1.5 统计方法

资料统计采用四格表χ^2^检验（包括精确χ^2^检验），统计软件SPSS 11.5。以*P*＜0.05为差异具有统计学意义。

## 2 结果

### 2.1 穿刺结果

75例患者共计穿刺177组病灶区域（表 1）。平均穿刺2.4组/例。全部177组穿刺区域，取影像学可见病变最大横截面积之短径计算，中位直径为18（5-50）mm，穿刺成功率为100.00%。平均穿刺时间为4.8（3-55）min，平均穿刺深度为28.4（15-40）mm。无操作相关并发症。

**1:** 75例患者淋巴结穿刺情况 Lymph node biospy conditions of 75 patient

Station	Samples (*n*)
2L	1
2R	28
4	10
4L	22
4R	45
7	40
8R	2
10L	4
10R	10
11L	1
11R	4
Tumor	10
Total	177

75例患者，送检细胞病理学75例，组织病理学37例，组织病理标本送检率49.33%。以177组穿刺区域计算，送检细胞病理学177例，组织病理学51例，组织病理标本送检率28.81%。

### 2.2 观察指标

#### 2.2.1 病理诊断

全部患者中，最终诊断阴性5例（结核3例，结节病2例），阳性70例。后者中6例肺内病灶为恶性病例，但5例淋巴结术后病理证实为反应性增生，1例淋巴结术后病理证实为囊肿。故实际穿刺纵隔病变阳性64例，阴性11例。

以最终确诊为“金标准”计算，EBUS-TBNA病理诊断准确率98.66%，敏感性98.43%，特异性100.00%，阳性预测值100.00%，阴性预测值91.67%。由于上述患者均CT诊断阳性，故对于CT病理病理诊断结果，未再进行上述统计（表 2）。

**2:** 75例患者CT及EBUS-TBNA病理确诊结果 Pathological diagnostic results of CT and EBUS-TBNA in 75 patients

CT	Final diagnosis		Total	EBUS-TBNA	Final diagnosis		Total
	+	-			+	-	
+	64	11	75	+	63	0	63
-	0	0	0	-	1	11	12
Total	64	11	75		64	11	75

#### 2.2.2 淋巴结转移诊断

以177组穿刺区域计算，CT诊断阳性161组，阴性16组。EBUS-TBNA诊断恶性病变136组，良性病变41组，后者中，31组纵隔镜或术后病理提示良性病变，最终诊断阴性。另10组出现于恶性病例中，以淋巴结大小判断，3例直径小于10 mm记为阴性，7例直径大于20 mm记为阳性。故最终诊断阳性143组，阴性34组。

以最终确诊为“金标准”计算，EBUS-TBNA病理诊断总体准确率为96.05%，敏感性为95.01%，特异性为100.00%，阳性预测值为100.00%，阴性预测值为82.93%。CT诊断准确率为78.51%，敏感性为93.01%，特异性为17.65%，阳性预测值为82.61%，阴性预测值为37.50%（表 3）。两者结果间除敏感性（χ^2^=0.56, *P*=0.435）外，均具有统计学差异（表 4）。

**3:** 177组淋巴结CT及EBUS-TBNA诊断结果 The diagnostic results of CT and EBUS-TBNA in 177 groups of lymph nodes

CT	Final diagnosis		Total	EBUS-TBNA	Final diagnosis		Total
	+	-			+	-	
+	133	28	161	+	136	0	136
-	10	6	16	-	7	34	41
Total	143	34	177		143	34	177

**4:** 177组淋巴结CT及EBUS-TBNA诊断结果比较 The diagnostic results comparison between CT and EBUS-TBNA in 177 groups of lymph nodes

	Statistical results		*χ*^2^	*P*
	CT (%)	EBUS-TBNA(%)		
Accuracy	78.53	96.05	22.91	< 0.001
Sensitivity	93.01	95.10	0.56	0.453
Specificity	17.65	100.00	44.26	< 0.001
Positive predictive value	82.61	100.00	24.12	< 0.001
Negative predictive value	37.50	82.93	-^a^	0.003
^a^: *fisher exact* *χ*^2^ test.				

#### 2.2.3 N分期

全部患者中，73例给与EBUS-TBNA分期。CT给予临床诊断N1为10例，N2为40例，N3为23例。经EBUS-TBNA检查后，19例（26.03%）出现分期改变，其中分期提高4例（5.48%），分期降低15例（20.55%，包括良性5例）（表 5）。

**5:** 73例患者CT及EBUS-TBNA检查N分期比较 N staging comparison between CT and EBUS-TBNA in 75 patients

Staging with CT (*n*)	Re-staging with EBUS-TBNA (*n*)
N1 (10)	N1 (7)
	N2 (2)
	N3 (1)
N2 (40)	Benigh (2)
	N0 (4)
	N1 (1)
	N2 (32)
	N3 (1)
N3 (23)	Benigh (3)
	N0 (2)
	N2 (3)
	N3 (15)

## 3 讨论

近年来，肺癌发病及死亡率在我国不断上升^[1, 2]^。随着各项诊疗技术的不断涌现，准确的诊断及治疗前分期已经被共认是制定综合治疗方案及推测预后的关键。在传统的纵隔淋巴结定性检查方法中，纵隔镜^[6]^是公认的“金标准”。但其诊断费用及创伤较大，涉及淋巴结区域多局限于N2/N3各组，且重复检查极为困难。因此，这一技术在国内目前尚未得到大规模的开展和应用。经气管镜穿刺技术（transbronchial needle aspiration，TBNA，无超声引导下的盲穿）在国内开展相对较多，但由于缺乏有效的实时监测，穿刺中对于周围组织尤其是血管的损伤一直令人担忧^[7]^。近年来，EBUS-TBNA以其技术操作简单、微创，涉及纵隔淋巴结区域广及可重复性强的优势，越来越多的得到临床的认可。

自EBUS-TBNA检查诞生之初到现在，有关安全性的报道一直令人满意，我们曾经的报道^[5]^和本次数据均显示，EBUS-TBNA属于一项安全的微创伤检查项目。并且随操作熟练度的增加，操作时间将明显减少^[5]^。

本组病例中，49.33%的患者及28.81%的病灶区域在穿刺中取得病理学标本。但是进一步的分析37例病例中，EBUS-TBNA病理学确诊恶性19例（51.35%），3例细胞较少无法诊断，15例组织病理学未见恶性证据（包括3例阴性病例）。EBUS-TBNA实际组织学确诊22例（59.46%）。以送检51组计算，病理确诊恶性29组（53.33%），细胞较少无法诊断6例，阴性结果16例（无实际阴性病例）。EBUS-TBNA实际组织学确诊29例（56.86%）。由于国内EBUS-TBNA检查使用的穿刺针为22号，其内径相对较细，因此，如何在EBUS-TBNA检查中留取足够多的标本用于组织病理学检测是目前的技术难点之一。究竟是何种原因导致组织病理学检查的困难，有待进一步研究证实。

有关EBUS-TBNA用于纵隔淋巴结诊断的报道中，多认为其具有较好的准确率，报道中其敏感性约89.0%-98.7%，特异性多数为100%^[5, 8-17]^，无论是与CT、PET-CT^[10-13]^或TBNA^[14]^相比，甚至是“金标准”纵隔镜^[3, 4]^相比，EBUS-TBNA似乎具有一定的优势。即使在化疗后^[15]^或肿瘤复发^[16]^的患者中，EBUS-TBNA的敏感性及特异性仍然保持在较高的水平。本组研究的结果与之相似。

目前主要存在的争议是，究竟EBUS-TBNA能否真正代替纵隔镜成为纵隔淋巴结定性检查的首选。由于伦理学的原因，头对头的前瞻性随机对照研究尚未见大范围报道，但部份已有研究已经提示，EBUS-TBNA的发展前景令人期待。Ernst等^[4]^报道一组66例患者研究结果提示，分组研究中，EBUS-TBNA检查较纵隔镜检查具有更高的诊断水平（91% *vs* 78%, *P*=0.007），进一步研究显示，这一差异主要来自于隆突下淋巴结（差异24%，*P*=0.011）。如果排除该淋巴结后，其它各组间未能显示进一步的统计学差异。同时，就患者而言，EBUS-TBNA与纵隔镜检查相比未能显示出明显的N分期差异性（93% *vs* 82%, *P*=0.083）。本组研究结果与之相似。由于本组患者的入组标准为CT怀疑肺癌淋巴结转移的患者，因此，敏感性检查未能出现明显的统计学差异。同时，由于病例数的关系，分组统计未能进行，以上结果尚待进一步的研究确定。同时由于EBUS-TBNA检察仍旧存在局限，尤其是对于5、6、8、9组淋巴结穿刺困难较大，因此单纯的EBUS-TBNA检察仍旧无法进行全面的淋巴结评价。Wallace等^[14]^指出，EBUS-TBNA联合EUS-FNA将能够较为全面的进行纵隔淋巴结评价，但由于目前对于EUS-FNA的报道尚不多见，进一步的深入研究希望在未来得以进行。

本组病例中，假阴性概率约5%左右，如何进一步减少穿刺的假阴性率是EBUS-TBNA检查将来的重点之一。Lee等^[18]^曾报道一组30例41组淋巴结穿刺结果显示，穿刺次数增加的同时，阳性预测值也随之增加，每组淋巴结穿刺3次时，阳性预测值达97.6%。因此增加穿刺次数将是进一步提高准确率的可能方法之一，究竟是否可行尚待证实。同时，提高穿刺针的直径将在理论上增加样本组织量，有助于提高诊断准确率，但由于设备开发的原因，其安全性及最终结果目前仍旧未见报道。

在肺癌的诊断工作中，在有效地进行分期诊断同时，EBUS-TBNA检查标本能否用于进一步的免疫组化及相关分子病理学研究也是普遍受到关注的热点之一。目前已经有肯定的报道^[19, 20]^，相信随着对这一方法研究的不断深入，将能够看到更好的结果。

EBUS-TBNA检查，技术操作简单、微创，涉及纵隔淋巴结区域广及可重复性强，其优势越来越多的得到临床的认可。我们有理由相信，在未来，其将对肺癌诊治工作产生积极的影响。
